# Osteometric data of Avian Fauna of Armenia: A baseline for zoologists and archaeozoologists

**DOI:** 10.1016/j.dib.2024.110059

**Published:** 2024-01-12

**Authors:** Luba Balyan, Nina Manaseryan, Mamikon Ghasabyan, Maria Kumelova, Andranik Gyonjyan

**Affiliations:** Institute of Zoology, Scientific Center of Zoology and Hydroecology, NAS Armenia, P. Sevak 7, Yerevan, 0014, Armenia

**Keywords:** Morphometrics, Birds, Bones, Comparative collection, Measurements

## Abstract

Modern bird skeletons stored in the faunal collections of the Institute of Zoology of the Scientific Center of Zoology and Hydroecology NAS Armenia constitute a source material for this dataset. The osteological material in the scientific collections has been accumulated in the course of faunal studies in Armenia over the span of 60 years. The osteometric dataset sheds light on the country's species diversity and includes cranial and postcranial measurements (carpometacarpus, humerus, tibia, femur, tarsometatarsus, radius and ulna) of 141 bird skeletons which belong to 81 bird species, 34 families and 17 orders.

Bird skeletons have been collected by means of specimen collection from the wild for scientific study prior to 1990s when the practice was common and recovered opportunistically after 1990s from birds found dead through natural causes and incidents.

Recent bird skeletons (bones) serve as a unique comparative resource for zoological research and for identifying bird bones recovered from archaeological and natural deposits.

Specifications Table

**Table:** 

Subject	Biological sciences/Zoology
Specific subject area	Morphometry of bird skeletons stored in the faunal scientific collections of the Institute of Zoology of Armenia
Data format	Raw Filtered
Type of data	Tables .xls format for the table dataset with labels and numbers Image 4 .tiff format images showing sampling sites
Data collection	Morphometric data were collected from scientific faunal collections stored at the Zoological Museum of the Institute of Zoology, within the Scientific Center of Zoology & Hydroecology Armenian NAS. It was stored in poor conditions for a lengthy time period but it was possible to recover. Each skeleton bone was labelled, measured with an accuracy of 0.5 mm using a Vernier caliper, and described to the extent possible (sex, location and time the specimens were collected).
Data source location	Institution: Scientific Center for Zoology and Hydroecology Armenian NAS, Institute of Zoology, Laboratory of Vertebrate Zoology, Yerevan, Armenia City: Yerevan Country: Armenia Sampling sites (locations): A total of 141 sampling locations span all of Armenia.
Data accessibility	Repository name: Mendeley Data identification number: 10.17632/8wfkb2mmns.1 Direct URL to data: https://data.mendeley.com/datasets/8wfkb2mmns/1 This dataset is curated by the Laboratory of Vertebrate Zoology of the Institute of Zoology, Scientific Center of Zoology & Hydroecology Armenian NAS

## 1 Value of the Data

• Osteometric data of birds facilitate comparative studies of avian bones measurements recovered from archaeological sites and natural deposits, allowing the observation of intraspecific variability and skeletal changes through time.
• Faunal collections serve as tangible, first-hand, documented evidence that can be used to study past bird distribution, migration routes.
• Archaeozoologists, ornithologists, anatomists, morphologists, paleontologists or researchers working in related disciplines will benefit from this dataset.

## 2 Data Description

The data article provides a description of the dataset of the linked repository which contains osteometric measurement data of modern bird skeletons deposited in the scientific collections of the Laboratory of Vertebrate Zoology the Institute of Zoology at the Scientific Center of Zoology and Hydroecology of NAS Armenia. The center's avian skeleton collection includes 165 specimens and is the largest avian osteology collection in Armenia. The information outlined in the database is not part of any published research article and is made available as a standalone data repository for scientists dealing with morphometric comparative studies of birds.

The linked Mendeley Data repository dataset contains an Excel file named ‘bird osteometrics’ with cranial and postcranial measurement data of modern bird specimens sampled over a period of 61 years (between 1961 and 2022). The featured measurement data is from 141 studied bird individuals which belong to 81 species, 34 families and 17 orders collected from 141 sampling points across all provinces of Armenia. Of these 141 individuals, 16 are female and 125 are male individuals. Table 1 provides an overview of the content of the linked dataset.

**Table 1:** Brief overview of the linked dataset (10.17632/8wfkb2mmns.1).

Item type	Title	Content description
Excel sheet (Table)	Bone measurements of bird skeletons stored in the scientific collection of the Laboratory of Vertebrate Zoology of the Institute of Zoology, the Scientific Center of Zoology and Hydroecology of NAS Armenia	Osteometric measurement data showing: a) order, family, species and sex of the bird specimen measured, b) bone measurements in mm and location where the bird skeletal material was sampled
Four Tiff image files	Map1.tiff Map2.tiff Map3.tiff Map4.tiff	Sampling locations of a selection of bird skeletons (or 81 out of 141)

Standard measurements (cranial and postcranial) were taken following the data collection procedure described by A. von den Driesch 1976 [3]. Because for most individuals the cranium and some of the postcranial skeletal elements were not available, Table 2 provides the exact set of measurements (abbreviations and definitions) used in the dataset as described by A. von den Driesch 1976 [3].

**Table 2:** Measurements used in the linked Mendeley dataset.

Bones	Quantity (N)	Measurement abbreviations	Definition of measurement zones
HUMERUS		GL	Greatest length
		Bp	Greatest breadth of proximal end
		SC	Smallest breadth of corpus
		Bd	Greatest breadth of distal end

ULNA		GL	Greatest length
		Bp	Greatest breadth of proximal end
		Dip	Diagonal of proximal end
		SC	Smallest breadth of corpus
		Did	Diagonal of distal end
RADIUS		GL	Greatest length
		SC	Smallest breadth of shaft
		Bd	Greatest breadth of distal end

CARPO-METACARPUS		GL	Greatest length
		L	Length of carpometacarpus II
		Bp	Greatest breadth of proximal end
		Did	Diagonal of distal end

FEMUR		GL	Greatest length
		Lm	Medial length
		Bp	Greatest breadth of proximal end
		Dp	Greatest depth of proximal end
		SC	Smallest breadth of corpus
		Bd	Greatest breadth of distal end
		Dd	Greatest depth of distal end

TIBIO-TARSUS		GL	Greatest length
		La	Axial length
		Dip	Diagonal of proximal end
		SC	Smallest breadth of corpus
		Dd	Greatest depth of distal end

TARSO-METATARSUS		GL	Greatest length
		Bp	Greatest breadth of proximal end
		SC	Smallest breadth of corpus
		Bd	Greatest breadth of distal end
CRANIUM		GL	Greatest length
		CBL	Condylobasal length
		GB	Greatest breadth
		GBP	Greatest breadth across the Processus postfrontales
		SBO	Smallest breadth between the orbits on the dorsal side
		GH	Greatest height in the median plane
		LP	Length from the Protuberantia occipitalis externa to the most aboral points of the processus frontales of the Incisivum in the mediam plane
		LI	(Greatest) length of the Incivisum

MANDIBLE		GL	Greatest length of one-half of the mandible
		LaF	Length from the most aboral point of the Facies articularis (articular surface) on one side to the Apex
		LS	Length of the Symphysis

Table 3 outlines the taxonomic list of birds for which the osteometric measurements is provided, number of individuals measured and geographic names of sampling locations. Because no geo-positioning technology was available in the early stages of material collection, names of the nearest settlements are used to describe the location of the sampling point.

**Table 3:** Bird skeleton list for which osteometric measurements are provided.

Order	Family	Species name	No. of individuals (n)	Location
1. Anseriformes	1. Anatidae	1. Gadwall – *Mareca strepera*	2	Surenavan, Jrarat
		2. Marbled Teal – *Marmaronetta angustirostris*	2	Surenavan, Tsovak
		3. Northern Pintail – *Anas acuta*	1	Armash
2. Galliformes	2. Phasianidae	4. Caucasian Grouse – *Lyrurus mlokosiewiczi*	3	Kalavan, Yeghipatrush, Katnarat
		5. Caspian Snowcock – *Tetraogallus caspius*	1	Rind
3. Phoenicopteriformes	3. Phoenicopteridae	6. Greater Flamingo – *Phoenicopterus roseus*	1	Karchaghbyur
4. Podicipediformes	4. Podicipedidae	7. Black-necked Grebe – *Podiceps nigricollis*	2	Armash, Norashen
5. Columbiformes	5. Columbidae	8. European Turtle Dove – *Streptopelia turtur*	3	Yeghegnadzor, Yerevan, Ashtarak
6. Cuculiformes	6. Cuculidae	9. Common Cuckoo – *Cuculus canorus*	2	Urut, Tandzut
7. Apodiformes	7. Apodidae	10. Alpine Swift – *Tachymarptis melba*	2	Gndevaz, Garni
		11. Common Swift – *Apus apus*	3	Abovyan, Yerevan, Yerevan
8. Gruiformes	8. Rallidae	12. Common Coot – *Fulica atra*	3	Ararat, Jrarat, Hayravank
9. Charadriiformes	9. Burhinidae	13. Eurasian Stone Curlew – *Burhinus oedicnemus*	1	Armavir
	10. Charadriidae	14. Little Ringed Plover – *Charadrius dubius*	3	Bagaran, Armavir, Jrarat
	11. Scolopacidae	15. Little Stint – *Calidris minuta*	3	Armash, Masis, Vardanashen
		16. Ruff – *Calidris pugnax*	3	Armash, Lchashen, Karchaghbyur
		17. Black-tailed Godwit – *Limosa limosa*	2	Armash, Norashen
		18. Green Sandpiper – *Tringa ochropus*	2	Armash, Garnarich
		19. Common Redshank – *Tringa totanus*	1	Armash
	12. Laridae	20. Armenian Gull – *Larus armenicus*	4	Yerevan, Sevan, Masis, Ardenis
		21. Common Tern – *Sterna hirundo*	2	Armash, Janfida
		22. Little Tern – *Sterna albifrons*	2	Armash, Getashen
10. Ciconiiformes	13. Ciconiidae	23. White Stork – *Ciconia ciconia*	1	Yeraskhahun
11. Pelecaniformes	14. Pelecanidae	24. Great White Pelican – *Pelecanus onocrotalus*	1	Ardenis
	15. Ardeidae	25. Eurasian Bittern – *Botaurus stellaris*	2	Yeraskhahun, Margara
		26. Purple Heron – *Ardea purpurea*	1	Ranchpar
		27. Great Egret – *Ardea alba*	1	Lchashen
12. Strigiformes	16. Strigidae	28. Eurasian Eagle Owl – *Bubo bubo*	2	Areni, Vedi
		29. Little Owl – *Athene noctua*	3	Vedi, Ptghni, Oshakan
13. Accipitriformes	17. Accipitridae	30. Griffon Vulture – *Gyps fulvus*	1	Anipemza
		31. Cinereous Vulture – *Aegypius monachus*	1	Urtsadzor
		32.Lesser spotted Eagle – *Clanga pomarina*	1	Fantan
		33. European Honey-buzzard – *Pernis apivorus*	3	Aralanj, Byurakan, Barzdrashen
		34. Steppe Eagle – *Aquila nipalensis*	2	Gorayk, Apnagyugh
		35. Golden Eagle – *Aquila chrysaetos*	1	Talin
		36. Booted Eagle – *Hieraaetus pennatus*	2	Amasia, Aghnjadzor
		37. Western Marsh-Harrier – *Circus aeruginosus*	1	Armash
		38. Montagu's Harrier – *Circus pygargus*	2	Gorayk, Musayelyan
		39. Black Kite – *Milvus migrans*	2	Yerevan, Yerevan
		40. Common Buzzard – *Buteo buteo*	1	Karbi
		41. Steppe Buzzard – *Buteo buteo menetriesi*	1	Horom
		42. Long-legged Buzzard – *Buteo rufinus*	2	Maralik, Arzni
		43. Levant Sparrowhawk – *Accipiter brevipes*	1	Garni
14. Bucerotiformes	18. Upupidae	44. Eurasian Hoopoe – *Upupa epops*	2	Yerevan, Ayntap
15. Coraciiformes	19. Meropidae	45. Blue-cheeked Bee-eater – *Merops persicus*	3	Armash, Armash, Surenavan
		46. European Bee-eater – *Merops apiaster*	2	Khachik, Armavir
16. Falconiformes	20. Falconidae	47. Common Kestrel – *Falco tinnunculus*	1	Urtsadzor
		48. Eurasian Hobby – *Falco subbuteo*	1	Gndevaz
17. Passeriformes	21. Laniidae	49. Red-backed Shrike – *Lanius collurio*	4	Lejan, Hatsik, Kechut, Surenavan
		50. Lesser Grey Shrike – *Lanius minor*	1	Vayk
17. Passeriformes	22. Corvidae	51. Eurasian Magpie – *Pica pica*	2	Yerevan, Byurehgavan
		52. Red-billed Chough – *Pyrrhocorax pyrrhocorax*	2	Shaghap, Voskevaz
		53. Alpine Chough – *Pyrrhocorax graculus*	2	Byurakan, Orgov
		54. Eurasian Jay – *Garrulus glandarius*	1	Bjni
		55. Rook – *Corvus frugilegus*	2	Ohanavan, Yerevan
		56. Carrion Crow – *Corvus corone*	2	Yerevan, Artashat
17. Passeriformes	23. Paridae	57. Great Tit – *Parus major*	1	Yerevan
17. Passeriformes	24. Alaudide	58. Turkestan's Short-toed Lark – *Alaudala heinei*	2	Armash, Ptghni
		59. Eurasian Skylark – *Alauda arvensis*	2	Lchashen, Arteni
		60. Horned Lark – *Eremophila alpestris*	4	Talin, Aniavan, Areni, Vayk
17. Passeriformes	25. Cinclidae	61. White-throated Dipper – *Cinclus cinclus*	1	Yerevan
17. Passeriformes	26. Tichodromidae	62. Wallcreeper – *Tichodroma muraria*	1	Vedi
17. Passeriformes	27. Sittidae	63. Eurasian Nuthatch – *Sitta europaea*	1	Ijevan
		64. Western Rock-nuthatch – *Sitta neumayer*	1	Surenavan
17. Passeriformes	28. Sturnidae	65. Rosy Starling – *Pastor roseus*	2	Areni, Bjni
		66. Common Starling – *Sturnus vulgaris*	1	Masis
17. Passeriformes	29. Turdidae	67. Ring Ouzel – *Turdus torquatus*	1	Arteni
		68. Fieldfare – *Turdus pilaris*	1	Solak
		69. Mistle Thrush – *Turdus viscivorus*	1	Byurakan
17. Passeriformes	30. Muscicapidae	70. Black Redstart – *Phoenicurus ochruros*	1	Yerevan
17. Passeriformes	31. Prunellidae	71. Radde's Accentor – *Prunella ocularis*	2	Nshkhark, Martiros
17. Passeriformes	32. Passeridae	72. Rock Sparrow – *Petronia petronia*	2	Garni, Goravan
		73. White-winged Snowfinch – *Montifringilla nivalis*	1	Byurakan
		74. House Sparrow – *Passer domesticus*	1	Yerevan
17. Passeriformes	33. Fringillidae	75. Eurasian Chaffinch – *Fringilla coelebs*	1	Nor Hachn
		76. Asian Crimson-winged Finch – *Rhodopechys sanguinea*	2	Byurakan, Paruyr Sevak
		77. Common Linnet – *Linaria cannabina*	1	Abovyan,
		78. Twite – *Linaria flavirostris*	1	Karaglukh
17. Passeriformes	34. Emberizidae	79. Corn bunting – *Emberiza calandra*	3	Ashtarak, Saravan, Lanjar
		80. Ortolan Bunting – *Emberiza hortulana*	1	Kaps
		81. Yellowhammer – *Emberiza citrinella*	1	Araks

The taxonomy and nomenclature used in Table 3 follow the Bird Life International Illustrated Checklist of the Birds of the World [5,6] and is based on the taxonomy published in the Handbook of the Birds of the World (HBW) [1].

Also included in the linked dataset are four image files in .Tiff format named ‘Map1.tiff’, ‘Map2.tiff’, ‘Map3.tiff’ and ‘Map4.tiff’ showing sampling locations at which the bird specimens have been obtained. When several specimens of the same species are available, the find location of one specimen, chosen arbitrarily, has been located on the map. The original images have a resolution of 300 dpi, with a width of 1500 pixels and a height of 1412 pixels.

‘Map1.tiff’ shows sampling locations for birds in the order *Anseriformes, Galliformes, Phoenicopteriformes, Podicipediformes, Columbiformes, Apodiformes* and *Gruiformes*. ‘Map2.tiff’ shows sampling locations for birds in the order *Charadriiformes, Ciconiiformes* and *Pelecaniformes*. ‘Map3.tiff’ shows sampling locations for birds in the order *Strigiformes, Accipitriformes, Bucerotiformes, Coraciiformes* and *Falconiformes*. ‘Map4.tiff’ shows sampling locations for birds in the order *Passeriformes* which is the largest on the list. Bird species shown on the map are numbered and color-coded according to their families and are presented on four separate maps in order to accommodate all data.

**Fig. 1:**
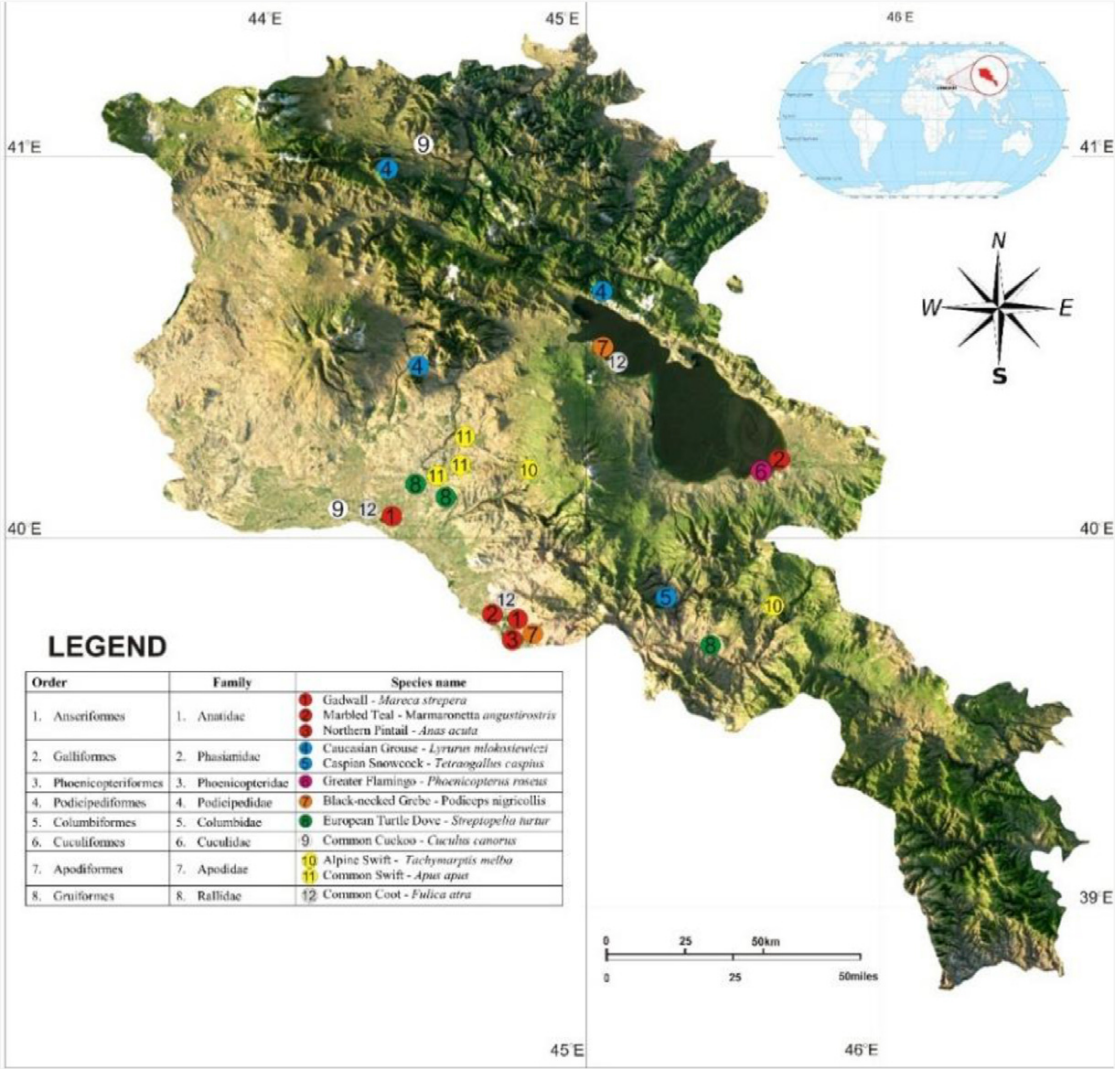
‘Map1.tiff’ displaying map of locations for birds in the order *Anseriformes, Galliformes, Phoenicopteriformes, Podicipediformes, Columbiformes, Apodiformes* and *Gruiformes.*

Geography of sampled skeleton material included in this manuscript covers all of Armenia with 141 sampling sites. Fig 1 shows one of the four maps - Map1.tiff with such sampling locations. Sampling sites are not geo-referenced because no geotracking devices were available in the early stages of material sampling and only locations are provided.

## 3 Experimental Design, Materials and Methods

### 3.1 Materials and methods

#### 3.1.1 Material

The measurement material used in the article includes cranial measurements of 83 skulls (cranium, mandible) and postcranial measurements of 141 skeletal parts (carpometacarpus, humerus, tibia, femur, tarsometatarsus, radius and ulna) from 81 bird species which belong to 34 families and 17 orders. The bird skeletons stored in the scientific collection funds of the Scientific Center for Zoology and Hydroecology NAS Armenia have been collected over the span of 60 years and stored with the aim to create a comparative skeletal collection fund and data for archaeological and zoological research.

Our choice of measurements of the sampled skeletal material relied on the preservation quality and state of the bones. Fractured and fragmented bones were excluded from the measurements. Despite no restrictions are set with regards to which skeletal parts should be measured [3,4], we used same measurements from each skeleton to achieve maximum comparability of the osteometric results.

Prior to 1990s avian skeletons were obtained by means of specimen collection from the wild for scientific study which was a common practice in the country. Starting from early 1990s the practice of amassing specimen collection for science has been restricted for ethical concerns and the present-day bird skeletons were collected opportunistically, involving the salvage of birds found dead through natural causes, confiscated from illegal hunting with the help of Environmental Inspectorate or collected from wildlife rescue centers.

#### 3.1.2 Location

Geographic area of sampling points of avian skeleton material collected over the period of 60 years cover all 11 administrative provinces of Armenia, including present-day protected areas (e.g. Lake Sevan National Park, Lake Arpi National Park). When a bird carcass has been found the original location where the distressed bird has been collected was used.

#### 3.1.3 Preparing bird specimens

The bird specimens obtained in the course of scientific field expeditions (until 1990) and those collected after that date (using opportunistic methods) have been meticulously treated in the laboratory using both mechanical and chemical preparation methods. Despite the methods of specimen collection have changed over time, the specimen preparation protocols remained the same. First of all, after careful examination and proper data recording the study specimens have been skinned (feathers and skin separated from the carcass) using a scalpel, whereby the tissues and the internal organs (muscles, brain, etc.) have been removed. This was followed by the removal of the excess tissues, ligaments and other pieces of meat and subsequently the cleaning of the skeletal material by simmering it in a pot of warm water to help tissue separation. Final degreasing was done using the 3 % hydrogen peroxide solution (H2O2). Subsequently the study skeletons have been washed and dried. Each skeletal material and skull has then been numbered, labelled and catalogued to maximize the scientific value of our osteometric studies. Sexing was done both visually (in case of sexually dichromatic species) and through examination of gonads [8].

#### 3.1.4 Bone measurements

Measurements of skeletal material were taken based on the references [2,3,7], using a vernier caliper with precision to 0.5 mm. Only absolute measurement data are provided. At least three bones were measured for each individual.

## Limitations

Our limitations in producing the current dataset referred to preservation quality and state of the bones. To this end, fractured and fragmented bones were excluded from the measurements and same measurements from each skeleton were used to ensure maximum comparability of the osteometric results.

## Ethics Statement

The authors have read and follow the ethical requirements for publication in Data in Brief and confirming that the current work does not involve human subjects, animal experiments, or any data collected from social media platforms.

## CRediT authorship contribution statement

**Luba Balyan:** Methodology, Writing – review & editing, Visualization. **Nina Manaseryan:** Conceptualization, Data curation, Writing – original draft. **Mamikon Ghasabyan:** Writing – review & editing. **Maria Kumelova:** Resources, Formal analysis, Visualization. **Andranik Gyonjyan:** Conceptualization, Investigation, Resources, Methodology, Data curation, Visualization.

## Data Availability

Osteometric Data of Avian Fauna of Armenia (Original data) (Mendeley Data)

## Appendix Supplementary materials

Image, application 1
